# The importance of universal child and family health services for equitable early development

**DOI:** 10.5694/mja2.70067

**Published:** 2025-09-30

**Authors:** Anna MH Price, Elodie O’Connor, Sharon R Goldfeld

**Affiliations:** ^1^ Centre for Community Child Health Royal Children’s Hospital Melbourne Melbourne VIC; ^2^ Murdoch Children’s Research Institute Melbourne VIC

**Keywords:** Primary care, Population health, Nursing, Child development

In Australia, growing political interest in the first 2000 days of life (from conception to five years) reflects the profound impact of early childhood on long term health, development and eventual societal participation.^1^ Recent national initiatives (eg, Early Years Strategy,^2^ Measuring What Matters framework^3^) and state‐level efforts (eg, New South Wales First 2000 Days Framework,^4^ Putting Queensland Kids First Plan^5^) seek a coordinated health and education system, prioritising prevention and early intervention. Broader early years advocacy includes a guarantee ensuring all children and families have access to the holistic supports necessary for optimal health and development.^6^

The best way to support children and families to thrive is by promoting positive experiences and preventing negative ones. Across Australian jurisdictions, child and family health (CFH; also abbreviated as CaFHS, or known as Child Health and Parenting Service [CHaPS], or as Maternal and Child Health [MCH]) services provide free, high quality, non‐stigmatising, well child health care from birth to school entry. As universal primary health care, they have a critical role in health promotion, prevention, and early identification, and should be accessible regardless of geographic location or socio‐economic status (Box 1).

Box 1 **Child and family health (CFH) services in Australia**

**Table:** 

Types of supports and services provided: ► Growth and developmental surveillance, immunisations, health education, mental health assessments for caregivers, addressing social determinants of health such as family violence, early intervention referrals, family‐centred care^8^, ^9^ Who provides them: ► Registered nurses with additional qualifications in midwifery or child and family health ► Aboriginal and/or Torres Strait Islander health workers and practitioners ► Social workers Key benefits: ► Building parenting capacity and confidence and connection with the service system ► Early detection and response to children’s developmental delays, growth concerns, parental mental health issues, and social risks, helping prevent more serious difficulties ► Reducing health disparities, particularly for groups who are often excluded or under‐represented Enhanced services: ► Additional CFH services for families experiencing more complex challenges ► Victoria provides up to 20 extra hours for families with children up to the age of three years experiencing difficulties such as poor parental mental health, family violence, or child protection concerns^10^ ► Some jurisdictions offer home visiting; for example, Queensland is rolling out the Maternal Early Childhood Sustained Nurse Home Visiting (MECSH) program in 2024–28, which is an Australian‐developed and effective model of nurse home visiting^11^

Despite growing political recognition of the importance of early childhood, Australia has primarily focused on early childhood education and care (ECEC); specifically, universal preschool at three and four years.^7^ CFH services are essential for engaging and supporting families from birth but have received less attention. Although a national framework was developed by state and territory representatives in 2011, it was not subsequently endorsed.^8^ As such, the sector lacks nationally consistent guidelines, as well as training and delivery standards.

This perspective article highlights CFH services as the health backbone — alongside ECEC — of a universal early childhood development system. It emphasises the importance of monitoring and equity in CFH services, and presents a pragmatic conceptual model for delivering universal, equitable CFH services in Australia.

## Author perspective

For 30 years, the Centre for Community Child Health (CCCH) has worked with families, communities, practitioners and policy makers to improve children’s health and development. As research and evaluation partners of CFH services, the authors are committed to strengthening CFH capacity and impact. CCCH partnerships include developing and evaluating CFH practice frameworks, supporting CFH providers in identifying early developmental risks, and supporting the implementation and evaluation of models of care that address the social determinants of health, such as sustained home visiting and Child and Family Hubs.

## The importance of monitoring and equity in child and family health services

Despite state and territory governments collecting data on CFH service delivery, public monitoring and data sharing remain limited. The few published studies highlight substantial variation in uptake. A 2024 study analysed CFH service use in NSW using data from 18 000 children born in 2014. Although guidelines recommend eight well child visits in the first 2000 days, 17% had no contact, 36% had 1–7 visits, 31% had 8–20 visits, and 17% had more than 20 visits, with some exceeding 100 contacts.^12^

Victoria’s CFH service system is among the most comprehensive, offering ten scheduled appointments from birth to school entry. Annual reporting data (published until 2018) showed that over 99% of families received a home visit within two weeks of birth, and attendance at the five scheduled appointments in the first six months exceeded 95%.^13^ This near‐universal reach was facilitated by legislation transferring birth notifications from maternity services to CFH services.

A cohort study in Melbourne tracked health service use via mothers’ daily diaries, finding that 98% of first time parents engaged with CFH services, averaging 14 CFH and ten general practice visits in the first postpartum year.^14^ More recent Victorian data (2019–21) from the School Entrant Health Questionnaire showed that around 70% of responding parents attended their child’s 3.5‐year CFH check, even during the COVID‐19 pandemic.^15^

Despite its universal design, CFH services are not consistently or equitably implemented, reflecting the inverse care law, where those who would benefit most from the highest quality services have the least access to them.^16^ In Victoria and Tasmania, women facing at least two socio‐economic or psychosocial adversities during pregnancy, such as poor mental health or smoking, had an average of 7.6 CFH visits by their child’s second birthday, similar to middle‐income cohorts.^11^, ^14^ Similarly, the 2024 study in NSW found no correlation between CFH visits and psychosocial risk.^12^ Research in Victoria has also revealed an inverse relationship between household income and nurse screening for family violence, despite lower income households experiencing higher rates of violence.^17^ Aboriginal and/or Torres Strait Islander families report lower engagement than non‐Indigenous families.^13^, ^18^

As Australia’s child population grows, children represent a smaller proportion of general practice visits,^19^ and a higher share of low urgency emergency department presentations.^20^ As most CFH data were collected before the COVID‐19 pandemic, it is difficult to assess recent trends in service reach. During the COVID‐19 pandemic, CFH services faced major constraints, particularly in Victoria and NSW, where extended lockdowns led to a heavy reliance on telehealth and to prioritisation of infants aged under eight weeks and of families experiencing adversity.^21^ A review by the Royal Children’s Hospital in Melbourne found a threefold increase in infant admissions for common well child concerns typically managed by CFH services, such as poor growth, feeding issues, irritability, and maternal mental health, compared with pre‐pandemic levels.^22^

## A conceptual model for delivering equitable child and family health services

Australia’s CFH services should serve as the health backbone of universal primary health care in the early years, delivered through proportionate universalism (also known as progressive universalism). This public health approach, adapted by Sir Michael Marmot, emphasises that improving health for all requires ensuring all families receive support, while providing tailored supports to those who would benefit from additional assistance:

To reduce the steepness of the social gradient in health [inequalities], actions must be universal, but with a scale and intensity that is proportionate to the level of disadvantage. We call this proportionate universalism.^23^

Proportionate universalism can be effectively embedded within existing CFH services, as illustrated in Box 2. This tiered model integrates evidence‐based approaches to identify and build on families’ existing capacity and strengths, balancing universal access with tailored support within the same service. CFH providers are trained to address concerns, reducing unnecessary referrals, long wait times, and the escalation of problems. This *seek and respond* model shifts away from a traditional *seek and refer* approach and aligns with the Australian Government’s Early Years Strategy and recently‐announced Thriving Kids plan (previously Foundational Supports).^2^, ^24^, ^25^ While the details of the latter are being designed, there is a clear need to focus on mainstream uplift to better identify and meet the needs to children and families with additional health and developmental concerns across the severity continuum.

Box 2 **Pragmatic conceptual model for equitable child and family health services**

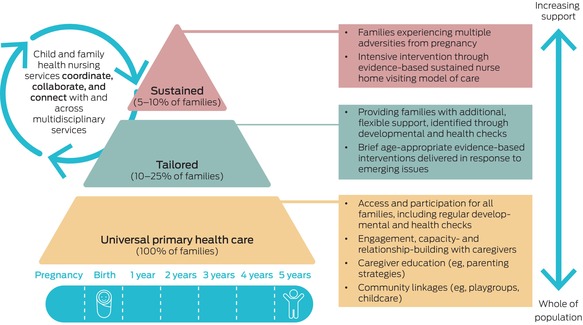

## Three tiers of proportionate universalism in child and family health

### Tier 1: universal support

The foundation of the model provides a universal platform for monitoring child development and identifying social factors that influence health. Relationship building with caregivers is central, ensuring engagement and early detection of concerns.

Ideally, the continuum of care would begin before birth. Pre‐ and peri‐conceptional care are critical periods during which support can influence outcomes for both parent and child. Strengthening integration between these services and CFH can help ensure smoother transitions and continuity of care across the first 2000 days.

### Tier 2: tailored support (10–25% of families)

Designed to provide families with additional, flexible support, this tier addresses caregiver concerns requiring more in‐depth exploration or brief evidence‐based interventions.^26^, ^27^ Examples include sleep, smoking cessation, maternal pain, and general developmental concerns. If an issue remains unresolved, caregivers may be referred to specialised services while continuing routine CFH care.

This middle tier is underdeveloped in Australia, and strengthening its evidence base will be key to refining its role and impact. This includes integrating effectively with general practice, paediatric, and allied services. This coordination ensures families receive comprehensive and continuous care, particularly when there are additional medical or developmental needs to respond to.

### Tier 3: sustained support (5–10% of families)

For families experiencing multiple adversities from pregnancy onwards, this tier offers intensive prevention through sustained home visiting,^26^, ^27^ a model of care backed by strong Australian and international evidence, making it one of the few public health programs with evidence for reducing inequities.^28^ International modelling demonstrates that the initial investment is recouped over the child’s lifetime, through improved health and developmental outcomes.^29^

### Strengthening child and family health services for equity and impact

For this conceptual model to function effectively, greater flexibility between tiers is essential, requiring:

- workforce planning to support scalable service delivery;
- adequate funding to sustain tailored interventions;
- structured and adaptable appointment guidelines to ensure consistent, high quality care; and
- ongoing data collection and quality improvement to track reach, outcomes and equity.

By embedding proportionate universalism within CFH services, Australia can maximise existing investments, enhance service impact, and improve health outcomes for all children and families, especially those who stand to benefit the most.

## Conclusion

CFH services are essential for promoting equitable health and developmental outcomes during the first 2000 days of life. They provide the health backbone of a universal early childhood development system, alongside education (ECEC). Despite their universal intent, inequities in access and inconsistent implementation across Australia limit their impact. A tiered, proportionate universalism model offers a pathway to better support diverse family capacity and strengths, from routine clinic‐based health promotion to sustained home visits. By optimising existing investments and leveraging Australia’s strong universal foundation, we can enhance CFH services and improve outcomes for all children and their families.

## Open access

Open access publishing facilitated by The University of Melbourne, as part of the Wiley – The University of Melbourne agreement via the Council of Australian University Librarians.

## Competing interests

No relevant disclosures.

## Provenance

Not commissioned; externally peer reviewed.

## Author contributions

Anna Price: Conceptualization, writing – original draft, writing – review and editing. Elodie O’Connor: Visualization, writing – original draft, writing – review and editing. Sharon Goldfeld: Conceptualization, supervision, writing – review and editing.

## References

[1] Moore T , Arefadib N , Deery A , West S . The first thousand days: an evidence paper. Melbourne: Centre for Community Child Health, Murdoch Children’s Research Institute; 2017. https://www.rch.org.au/uploadedFiles/Main/Content/ccchdev/CCCH‐The‐First‐Thousand‐Days‐An‐Evidence‐Paper‐September‐2017.pdf (viewed Sept 2025).

[2] Australian Government , Department of Social Services. Early Years Strategy. Canberra: Commonwealth of Australia, 2023. https://www.dss.gov.au/families‐and‐children‐programs‐services/early‐years‐strategy (viewed Mar 2024).

[3] Australian Government. Measuring What Matters — Australia’s First Wellbeing Framework . Canberra: Australian Government, 2023. https://treasury.gov.au/publication/p2023‐mwm (viewed July 2025).

[4] New South Wales Health . The First 2000 Days Framework. Sydney: NSW Government, 2019. https://www1.health.nsw.gov.au/pds/Pages/doc.aspx?dn=PD2019_008 (viewed Sept 2025).

[5] Queensland Government . Putting Queensland Kids First: giving our kids the opportunity of a lifetime. Brisbane: State of Queensland, Department of the Premier and Cabinet; 2024. https://www.qld.gov.au/about/putting‐qld‐kids‐first (viewed Sept 2025).

[6] European Commission . Employment, Social Affairs and Inclusion; Investing in children. European Child Guarantee [website]. https://ec.europa.eu/social/main.jsp?catId=1428&langId=en (viewed Mar 2023).

[7] Australian Government Department of Education . Building a universal early education and care system. Canberra: Commonwealth of Australia, 2024. https://www.education.gov.au/early‐childhood/announcements/building‐universal‐early‐education‐and‐care‐system (viewed Sept 2025).

[8] Schmied V , Kruske S , Barclay L , Fowler C . National Framework for Universal Child and Family Health Services. Canberra: Commonwealth of Australia, 2011. https://www.health.gov.au/sites/default/files/2023‐01/national‐framework‐for‐universal‐child‐and‐family‐health‐services.pdf (viewed July 2025).

[9] McLean K , Goldfeld S , Molloy C , et al. Screening and surveillance in early childhood health: rapid review of evidence for effectiveness and efficiency of models. Sydney: The Sax Institute, 2014. https://www.health.nsw.gov.au/kidsfamilies/MCFhealth/Documents/screening‐and‐surveillance‐in‐early‐childhood.pdf (viewed July 2025).

[10] Victorian Department of Health and Human Services . Enhanced maternal and child health program guidelines. Melbourne: State of Victoria, 2019. https://www.health.vic.gov.au/publications/enhanced‐maternal‐and‐child‐health‐program‐guidelines (viewed Sept 2025).

[11] Price A , Bryson H , Mensah FK , et al. Embedding nurse home visiting in universal healthcare: 6‐year follow‐up of a randomised trial. Arch Dis Child 2023; 108: 824‐832. 37399321 10.1136/archdischild-2023-325662

[12] Kemp L , Donohoe K , Matthews P , Aspery W . Translating “proportionate universal healthcare” into meaningful system design to optimize equity in child and family services. J Adv Nurs 2024; 10.1111/jan.16298 [Epub ahead of print]. PMC12535313 38922956

[13] Victorian Government Department of Health . Maternal Child and Health reporting and data. Melbourne: State of Victoria, 2025. https://www.health.vic.gov.au/maternal‐child‐health/maternal‐child‐and‐health‐reporting‐and‐data (viewed Dec 2024).

[14] Goldfeld SR , Wright M , Oberklaid F . Parents, infants and health care: utilization of health services in the first 12 months of life. J Paediatr Child Health 2003; 39: 249‐253. 12755928 10.1046/j.1440-1754.2003.00146.x

[15] Victorian State Government , Department of Education and Training. State findings from the School Entrant Health Questionnaire. Melbourne: State of Victoria, 2021. https://www.vic.gov.au/school‐entrant‐health‐questionnaire (viewed Jan 2025).

[16] Mercer SW , Patterson J , Robson JP , et al. The inverse care law and the potential of primary care in deprived areas. Lancet 2021; 397: 775‐776. 33640047 10.1016/S0140-6736(21)00317-2

[17] Hooker L , Taft A . Who is being screened for intimate partner violence in primary care settings? Secondary data analysis of a cluster randomised trial. Matern Child Health J 2021; 25: 1554‐1561. 33954881 10.1007/s10995-021-03136-0

[18] Austin C , Arabena K . Improving the engagement of Aboriginal families with maternal and child health services: a new model of care. Public Health Res Pract 2021; 31: 30232009. 34104933 10.17061/phrp30232009

[19] Freed GL , Spike NA , Sewell JR , et al. Changes in longer consultations for children in general practice. J Paediatr Child Health 2013; 49: 325‐329. 23517187 10.1111/jpc.12157

[20] Freed G , Gafforini S , Carson N . Age‐related variation in primary care‐type presentations to emergency departments. Aust Fam Physician 2015; 44: 584‐588. 26510148

[21] Adams C , Ridgway L , Hooker L . Maternal, child and family nursing in the time of COVID‐19: the Victorian Maternal and Child Health Service experience. Australian Journal of Child and Family Health Nursing 2020; 17: 12‐15.

[22] Licheni SH , Devaraja L , Watson B , et al. Impact of COVID‐19 public health restrictions on hospital admissions for young infants in Victoria, Australia. J Paediatr Child Health 2022; 58: 1001‐1006. 35020962 10.1111/jpc.15885

[23] Marmot M , Atkinson T , Bell J , et al. Fair society, healthy lives: the Marmot Review. A strategic review of health inequalities in England post‐2010. London: Marmot Review, 2010. https://www.instituteofhealthequity.org/resources‐reports/fair‐society‐healthy‐lives‐the‐marmot‐review/fair‐society‐healthy‐lives‐full‐report‐pdf.pdf (viewed Sept 2025).

[24] Australian Government Department of Social Services . Foundational Supports consultation. https://engage.dss.gov.au/foundational‐supports/ (viewed Dec 2024).

[25] Australian Government , Department of Health, Disability and Ageing. Thriving Kids — a new program to support children with developmental delay and autism, and their families. Canberra: Commonwealth of Australia, 2025. https://www.health.gov.au/news/thriving‐kids‐a‐new‐program‐to‐support‐children‐with‐developmental‐delay‐and‐autism‐and‐their‐families (viewed Sept 2025).

[26] Goldfeld S , D’Abaco E , Bryson H , et al. Surveying social adversity in pregnancy: the antenatal risk burden experienced by Australian women. J Paediatr Child Health 2018; 54: 754‐760. 29442394 10.1111/jpc.13860

[27] Sawyer AC , Gialamas A , Pearce A , et al. Five by Five: a Supporting Systems Framework for Child Health and Development. Adelaide: University of Adelaide, 2014. https://health.adelaide.edu.au/betterstart/system/files/media/documents/2022‐02/five‐by‐five‐dec‐2014.pdf (viewed July 2025).

[28] Zimmerman FJ . Population health science: fulfilling the mission of public health. Milbank Q 2021; 99: 9‐23. 33320388 10.1111/1468-0009.12493 PMC7984664

[29] Miller TR . Cost–benefit analysis of the Nurse–Family Partnership Program, final report to Pew Charitable Trust. Calverton (MD): Pacific Institute for Research and Evaluation, 2012. https://www.researchgate.net/publication/332233483_Cost‐Benefit_Analysis_of_the_Nurse‐Family_Partnership_Program_Final_Report_to_the_Pew_Charitable_Trust (viewed Apr 2024).

